# Brentuximab vedotin in relapsed/refractory Hodgkin lymphoma. The Hellenic experience

**DOI:** 10.1002/hon.2383

**Published:** 2017-02-20

**Authors:** Maria K. Angelopoulou, Theodoros P. Vassilakopoulos, Ioannis Batsis, Ioanna Sakellari, Konstantinos Gkirkas, Vasiliki Pappa, Panagiota Giannoulia, Ioannis Apostolidis, Christos Apostolopoulos, Paraskevi Roussou, Panayiotis Panayiotidis, Maria Dimou, Marie‐Christine Kyrtsonis, Maria Palassopoulou, Georgios Vassilopoulos, Maria Moschogiannis, Christina Kalpadakis, Dimitrios Margaritis, Alexander Spyridonidis, Eurydiki Michalis, Konstantinos Anargyrou, Panagiotis Repousis, Eleutheria Hatzimichael, Zoi Bousiou, Elias Poulakidas, Dimitrios Grentzelias, Nikolaos Harhalakis, Gerassimos A. Pangalis, Achilles Anagnostopoulos, Panagiotis Tsirigotis

**Affiliations:** ^1^ Department of Hematology, Laikon General Hospital National and Kapodistrian University of Athens Athens Greece; ^2^ Hematology and Bone Marrow Transplantation Department General Hospital of Thessaloniki Papanikolaou Thessaloniki Greece; ^3^ 2nd Department of Internal Medicine, Faculty of Medicine, ATTIKON General University Hospital National and Kapodistrian University of Athens, Medical School Athens Greece; ^4^ Department of Hematology Evaggelismos General Hospital Athens Greece; ^5^ Third Department of Medicine, “Sotiria” General Hospital of Thoracic Diseases, Hematology Unit National and Kapodistrian University of Athens, Medical School Athens Greece; ^6^ 1st Department of Propedeutic Internal Medicine, Laikon General Hospital National and Kapodistrian University of Athens, Medical School Athens Greece; ^7^ Department of Hematology, Larissa University Hospital University of Thessalia Larissa Greece; ^8^ Department of Hematology Athens Medical Center Athens Greece; ^9^ Department of Hematology, Heraklion University Hospital University of Crete Heraklion Greece; ^10^ Department of Hematology Democritus University of Thrace Medical School Alexandroupolis Greece; ^11^ Bone Marrow Transplantation Unit University of Patras Patras Greece; ^12^ Department of Clinical Hematology “G.Gennimatas” Athens General Hospital Athens Greece; ^13^ Department of Hematology Air Force Hospital Athens Greece; ^14^ Department of Hematology Metaxa Hospital Pireaus Greece; ^15^ Department of Hematology, Ioannina University Hospital University of Ioannina Ioannina Greece; ^16^ Department of Hematology 401 Military Hospital of Athens Athens Greece; ^17^ Hygeia General Hospital Athens Greece

**Keywords:** autologous stem cell transplantation, brentuximab vedotin, Hodgkin lymphoma, prognostic factors, relapsed/refractory

## Abstract

This retrospective study aimed to describe the Hellenic experience on the use of brentuximab vedotin (BV) in relapsed/refractory (R/R) Hodgkin lymphoma (HL) given within its indication. From June 2011 to April 2015, ninety‐five patients with R/R HL, who received BV in 20 centers from Greece, were analyzed. Their median age was 33 years, and 62% were males. Sixty‐seven patients received BV after autologous stem cell transplantation failure, whereas 28 patients were treated with BV without a prior autologous stem cell transplantation, due to advanced age/comorbidities or chemorefractory disease. The median number of prior treatments was 4 and 44% of the patients were refractory to their most recent therapy. The median number of BV cycles was 8 (range, 2‐16), and the median time to best response was the fourth cycle. Fifty‐seven patients achieved an objective response: twenty‐two (23%), a complete response (CR), and 35 patients (37%), a partial, for an overall response rate of 60%. Twelve patients (13%) had stable disease, and the remaining twenty‐six (27%) had progressive disease as their best response. At a median follow‐up of 11.5 months, median progression‐free survival and overall survival were 8 and 26.5 months, respectively. Multivariate analysis showed that chemosensitivity to treatment administered before BV was associated with a significantly increased probability of achieving response to BV (*P* = .005). Bulky disease (*P* = .01) and response to BV (*P* <.001) were significant for progression‐free survival, while refractoriness to most recent treatment (*P* = .04), bulky disease (*P* = .005), and B‐symptoms (*P* = .001) were unfavorable factors for overall survival. Among the 22 CRs, 5 remain in CR with no further treatment after BV at a median follow‐up of 13 months. In conclusion, our data indicate that BV is an effective treatment for R/R HL patients even outside clinical trials. Whether BV can cure a fraction of patients remains to be seen.

## 1 INTRODUCTION

Salvage chemotherapy followed by high‐dose therapy and autologous hematopoietic stem cell transplantation (ASCT) is the treatment of choice for relapsed/refractory (R/R) Hodgkin lymphoma (HL) patients.^1^, ^2^, ^3^ This therapeutic strategy can provide long‐term disease control in approximately 50% of R/R patients.^4^, ^5^ For patients who relapse after ASCT, conventional chemotherapy options are usually unsatisfactory, and their outcome is rather dismal with a median overall survival (OS) of 2 years.^6^, ^7^ Relapsed disease after ASCT is considered incurable, unless allogeneic transplantation is applied. However, very few patients can achieve this goal, since refractory disease often hampers the benefit of this procedure.

Brentuximab vedotin (BV) is an antibody‐drug conjugate targeting the CD30 antigen expressed on the surface of the malignant cells and leading to G2/M cell cycle arrest through disruption of the microtubule network.^8^, ^9^ A multicenter phase II trial of BV in patients with HL recurring after ASCT demonstrated an overall response rate (ORR) and complete response (CR) rate of 75% and 34%, respectively,^10^ with a median progression‐free survival (PFS) extending to 9.3 months.^11^ Based on this study, accelerated approval was granted by the US Food and Drug Administration in 2011 to BV, while the European Medicines Agency approved BV in October 2012 for R/R CD30+ HL patients following ASCT or following at least 2 prior therapies when ASCT or multiagent chemotherapy is not a treatment option.

Herein, we present the Hellenic experience with BV in patients with R/R HL, outside clinical trials, reflecting everyday clinical practice. Furthermore, we wanted to report the pattern of its use, to identify possible prognostic factors and investigate whether a fraction of patients can achieve long‐term disease control with BV as the sole treatment.

## 2 METHODS

This was a retrospective multicenter study among 20 centers in Greece aiming to collect data on patients with R/R HL treated with BV. Between June 2011 and April 2015, one hundred patients received BV within its indication. The study was approved by the Institutional Review Board of the participating hospitals. All patients had histologically confirmed R/R CD30+ HL and had received at least 2 cycles of BV, either due to disease progression after ASCT or after at least 2 prior therapies if ASCT was not indicated because of advanced age, insufficient stem cell collection, or chemoresistant disease. Two coordinating centers (Laikon and Attikon Hospitals) collected the data through a detailed form completed by the treating physicians and reviewed by the coordinators (MKA, PT). Five patients were excluded from this analysis, 3 due to insufficient data and 2 due to a histologic diagnosis of gray‐zone lymphoma. Thus, 95 patients were finally analyzed.

BV was administered as a 30‐minute infusion at the dose of 1.8 mg/kg of body weight every 3 weeks for a maximum of 16 cycles. The dose was capped to 180 mg for patients over 100 kg. Primary refractory disease was defined as no CR or relapse within 3 months of first line therapy. Early and late relapse were defined as relapse within or beyond 12 months after the end of first‐line treatment, respectively. Bulky disease was defined as a mass measuring >10 cm in its transverse diameter.

All patients underwent baseline assessments including physical examination, routine laboratory tests, and radiological examinations prior to BV. Response assessment was based on the revised response criteria for malignant lymphoma.^12^ However, radiologic tests were not centrally reviewed for a strict definition of response to be applicable. The treating physicians used computed tomography and/or positron emission tomography/computed tomography scans for response assessment according to their practice and test availability. The best response and the cycle after which best response was documented were recorded. The primary endpoint of the study was PFS, whereas ORR and OS were secondary endpoints.

### 2.1 STATISTICS

PFS was defined as the time from BV initiation to progression, relapse, or death of any cause. For PFS estimation, patients without disease progression after BV were censored at the time of last follow‐up or at the time of subsequent treatment. OS was measured from the time of BV initiation to death of any cause. PFS and OS along with 2‐sided 95% confidence interval (CI) were estimated using the Kaplan‐Meier method.^13^ Survival functions were compared using the log‐rank test. Significant variables at *P* < .05 in the univariate analyses were evaluated in multivariate analyses. The following covariates were entered in the multivariate analysis model: age at BV initiation, gender, number of treatments administered before BV (<3 vs > 3), response to initial treatment (primary refractory disease vs early relapse vs late relapse), response to the last chemotherapy regimen administered before BV (chemosensitive vs chemorefractory), disease stage (I/II vs III/IV), bulky disease, extranodal involvement and B‐symptoms at BV initiation, previous ASCT, and response to BV (CR vs partial response [PR]) vs no response]. Multivariate analysis for response to BV was performed by using a multiple logistic regression model, while for PFS and OS, a Cox proportional hazard model was used. Analysis of the data was perfomed using Medcalc and R software.

## 3 RESULTS

### 3.1 Patients' characteristics

Patients' median age at BV initiation was 33 years. More than half of them were primary refractory to first line treatment, whereas 19% and 24% had early and late relapse, respectively. Among the 95 patients, 67 received BV after ASCT failure, whereas in 20, BV was administered as salvage chemotherapy due to resistant disease with the intention to proceed to ASCT. Eight patients were considered ineligible for ASCT due to advanced age and/or poor performance status and received BV as third or more salvage. At the time of BV initiation, 2/3 had advanced disease stage, more than 1/3 had B‐symptoms, and almost half of them had extranodal involvement. In addition, 15% had bulky disease. The median time from diagnosis and ASCT to BV initiation was 35 months (6‐287) and 18 months (3‐108), respectively. The median time from the first documented relapse post ASCT to BV initiation was 8 months (range, 1‐81). Table 1 depicts patients' characteristics from the pivotal phase II study and 5 other published series of patients reporting their national experience in comparison to ours.

**Table 1:** Patients' characteristics from the present analysis in comparison with the pivotal phase II study and other published series

Characteristics	Phase‐II Study	Germany	Italy	Asia	Turkey	France	Greece
Number of points	102	45	65	22	58	240	95
Male sex, %	53	49	52	68	64	65	62
Primary refractory disease	71	62	69	55	49	49	57
Prior ASCT, %	100	87	88	77	80	59	70
Prior allo‐SCT, %	0		4.6		2	15	2.1
Median number of prior treatments (range)	3.5 (1‐13)	4 (2‐12)	4 (2‐13)	NR	4 (2‐7)	3 (1‐13)	4 (1‐9)
Refractory to prior last treatment, %	42	64	80	NR	72	56	44
Median time (months) from diagnosis to BV treatment (range)	40 (12‐220)	48 (10‐180)	NR	41 (14‐199)	NR	31 (3‐336)	35 (6‐287)
Disease characteristics at BV treatment:							
Advanced clinical stage/B‐symptoms, %	NR/NR	73/ 44	NR/45	95/68	78/47	NR/NR	63/36
Bulky disease, %	NR	4^a^	NR	NR	NR	NR	15
Extranodal involvement, %	NR	73	NR	64	NR	NR	45
ECOG PS ≤1, %	100	82	77	45	80	NR	NR

Abbreviations: allo‐SCT indicates allogeneic stem cell transplantation; ASCT, autologous stem cell transplantation; BV, brentuximab vedotin; NR, not reported; PS, performance status.

a Bulky mediastinum.

### 3.2 Response to BV

The median number of BV cycles was 8 (range, 2‐16). The exact timing of response assessment was not predefined. However, the median cycle number for best response achievement was the fourth (range, 2‐12).

Among 95 patients, 57 achieved an objective response: twenty‐two (23%), a CR, and 35 patients (37%), a PR, for an ORR of 60%. Twelve patients (13%) had stable disease and the remaining twenty‐six (27%) had progressive disease (PD) as their best response. Table 2 depicts efficacy of BV in published series including the present study.

**Table 2:** Outcome after BV in comparison with the pivotal phase II study and other published series

Characteristics	Phase‐II Study	Germany	Italy	Asia	Turkey	France	Greece
BV cycles: # (range)	9 (1‐16)	7 (1‐12)	8 (3‐16)	5 (1‐18)	7 (2‐18)	6 (1‐16)	8 (2‐16)
Cycle of response evaluation	2, 4, 7, 10, 13, and 16	NR	3 and 8	every 1‐2 cycles	2, 5, and ≥6	4	3 or 4
Median time to response	5.7 wks^a^	NR	NR	0.9 mo	NR	4 cycles	4 cycles
ORR/CR, %	*75/34*	*60/22*	*71/22*	*73/18*	*64/27*	*60/34*	*60/23*
SD, %	22	29	17	18	8	*8*	13
PD, %	3	11	12	5	29	*28*	27
Median time of follow‐up (months)	33	NR	13.2	NR	NR	16	11.5
median PFS (months)	9.3	8	6.8	5.7	7	6.8	8
median OS (months)	40.5	NR	NR	NR	NR	NR	26.5
OS, %	3 y, 47	1 y, 83	1 y, 75	1 y, 67	1 y, 71	2 y, 58	2 y, 67

a Median time to CR: 12 weeks.

Abbreviations: BV, brentuximab vedotin; CR, complete response; NR, not reported; ORR, overall response rate; OS, overall survival; PD, progressive disease; PFS, progression‐free survival; SD, stable disease.

Prognostic factor univariate analysis for response revealed that less pretreated patients (<3 prior treatments), with nonbulky disease, the ones who received BV after ASCT and those who were chemosensitive to their last prior treatment to BV responded significantly better (Table 3). By multivariate analysis, only chemosensitivity to last prior treatment remained significant (*P* = .005): Patients who were sensitive to their last prior chemotherapy achieved an ORR of 75% vs 40% for those who were chemorefractory (Table 4).

**Table 3:** Univariate prognostic factor analysis

	Response		PFS		OS	
	ORR, %	*P*	Median (Months)	*P*	Median (Months)	*P*
Characteristic
Sex (female vs male)	62 vs 59	NS	9 vs 6	NS	28 vs 27	.049
B‐symptoms (no vs yes)	64 vs 53	NS	8 vs 6	NS	26 vs 16	<.001
Number of previous treatments (≤3 vs >3)	69 vs 49	.058	9 vs 5	.01	NR vs 27	NS
Refractory to most recent treatment (no vs yes)	75 vs 40	<.001	9 vs 4	.005	28 vs 26	.008
Bulky disease (no vs yes)	65 vs 21	.014	9 vs 3	<.001	27 vs 10	<.001
Extranodal involvement (no vs yes)	67 vs 55	NS	9 vs 6	NS	27 vs 26	.057
Prior ASCT (yes vs no)	69 vs 39	.009	9 vs 4	.007	27 vs NR	NS
Response to BV (yes vs no)	NA	NA	12 vs 3	<.001	28 vs 26	NS
CR to BV (yes vs no)	NA	NA	14 vs 4	<.001	NR vs 26	.014

Abbreviations: ASCT indicates autologous stem cell transplantation; BV, brentuximab vedotin; CR, complete response; NA, not applicable; NR, not reached; NS, nonsignificant; ORR, overall response rate; OS, overall survival; PFS, progression‐free survival.

**Table 4:** Multivariate prognostic factor analysis

Response to BV (multiple logistic regression)			
Covariate	Odds Ratio	95% CI	*P*
Response to previous treatment before BV (Chemosensitive vs chemorefractory)	0.22	0.07‐0.65	.005

Progression‐Free Survival (Cox proportional hazard analysis)			
Covariate	Hazard Ratio	95% CI	
Bulky disease (yes vs no)	2.28	1.18–4.38	.01
Response to BV (yes vs no)	0.08	0.04–0.16	<.001

Overall Survival (Cox proportional hazard analysis)			
Covariate	Hazard Ratio	95% CI	*P*
Response to previous treatment before BV (Chemosensitive vs chemorefractory)	0.32	0.10–0.98	.04
Bulky disease (yes vs no)	4.62	1.57–13.64	.005
B‐symptoms (yes vs no)	5.52	1.95–15.62	.001

Abbreviation: BV indicates brentuximab vedotin.

### 3.3 Progression‐free survival and Overall survival

**Figure 1:**
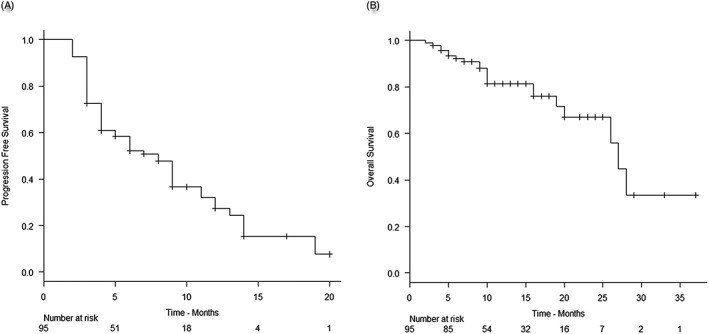
Treatment outcome with brentuximab vedotin: A, Progression‐free survival. B, Overall survival.

At a median follow‐up time of 11.5 months, disease progression was observed in 62 of 95 patients, while 21 of 95 patients expired after treatment with BV. The median PFS and OS were 8 months (95% CI, 5‐9) and 26.5 months (95% CI, 20‐31), respectively, with a 2‐year OS reaching 67% (Figure 1).

**Figure 2:**
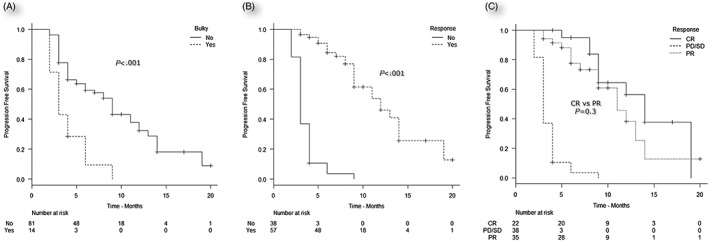
Prognostic factors for progression‐free survival: A, Bulky disease at BV initiation, (yes vs no). B, Response to treatment with BV, (yes vs no). C, Type of response to brentuximab vedotin (CR vs PR vs PD/SD). CR indicates complete response; PD, progressive disease; PR, partial response; SD, stable disease

For PFS, the following factors proved significant by univariate analysis: number of prior treatments (<3 vs >3), refractoriness to most recent treatment, bulky disease at initiation of BV, prior ASCT, and response to BV (Table 3). By multivariate analysis, the factors that remained significant for PFS were bulky disease (*P* = .01) and response to BV (*P* < .001) (Table 4). The median PFS for patients with bulky was 3 vs 9 months for those with nonbulky disease (Figure 2A). Responders to BV proved to have a significantly superior prognosis with a median PFS of 12 months compared with 3 months for nonresponders (Figure 2B). PFS did not differ significantly between complete and partial responders (median PFS, 14 and 11 months, respectively, *P* = non‐significant, Figure 2C).

**Figure 3:**
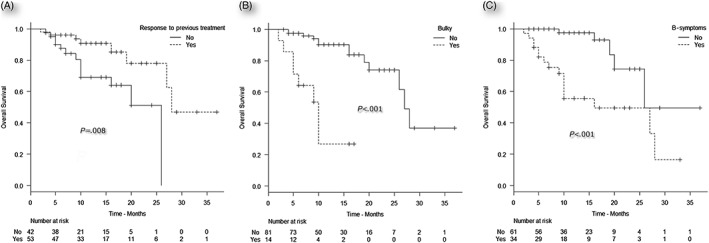
Prognostic factors for overall survival: A, Response to most recent treatment prior to brentuximab vedotin (BV; chemosensitive vs chemorefractory). B, Bulky disease at BV initiation, (yes vs no). C, Presence of B‐symptoms at BV initiation (yes vs no). CR indicates complete response; PD, progressive disease; PR, partial response; SD, stable disease

Male gender, refractoriness to most recent chemotherapy prior to BV, bulky disease, extranodal involvement, B‐symptoms, and failure to achieve CR with BV were identified as poor prognostic factors for OS by univariate analysis (Table 3). Factors that remained significant for OS by multivariate analysis were refractoriness to most recent treatment (*P* = .04), bulky disease (*P* = .005), and B‐symptoms (*P* = .001) (Table 4). Median OS for nonresponders to the last previous treatment before BV was 26 months for non‐responders vs 28 for responders. Patients with bulky disease and B‐symptoms had a median OS of 10 and 16 months, respectively compared with 27 and 26 months for those with nonbulky disease and absence of B‐symptoms, respectively (Figure 3).

### 3.4 Treatment after BV

Among the 95 patients included in the study, 67 received BV after ASCT and 28 without a preceding ASCT. Among the 67 patients who received BV after ASCT, 22 received further chemotherapy for subsequent relapse/progression, 18 underwent allogeneic stem cell transplantation (allo‐SCT), 22 received no further treatment, 2 were treated with radiotherapy, and 1 with a second ASCT, while the remaining 2 patients were still under BV treatment at the time of the present analysis. Among the 18 patients who underwent allo‐SCT after BV, the majority (14 of 18) were alive without evidence of active disease. On the contrary, among the ones who received subsequent chemotherapy and those who did not receive any further treatment, only 2 of 22 and 8 of 22, respectively, had not developed PD at the time of the present analysis.

There were 28 patients who received BV without a previous ASCT. Among these, 8 patients were not eligible for ASCT due to advanced age or poor performance status, while in the remaining 20 patients, BV was administered in an effort to achieve disease control with intent to proceed to ASCT. Eight of these 20 patients did not have an objective response to BV and were considered as noneligible for auto‐SCT by the treating physician, while 2 other patients who achieved CR and PR after BV refused any further treatment. Therefore, BV was used as a bridge to ASCT in 10 of 20 patients. They received a median number of 4 cycles before ASCT, and 3 of 10 achieved a response (PR). Six of them are currently alive without evidence of disease at a median time of 8 months (3‐17) after ASCT, while 4 progressed shortly thereafter (between 2 and 6 months post ASCT).

### 3.5 Outcome of complete responders to BV

**Figure 4:**
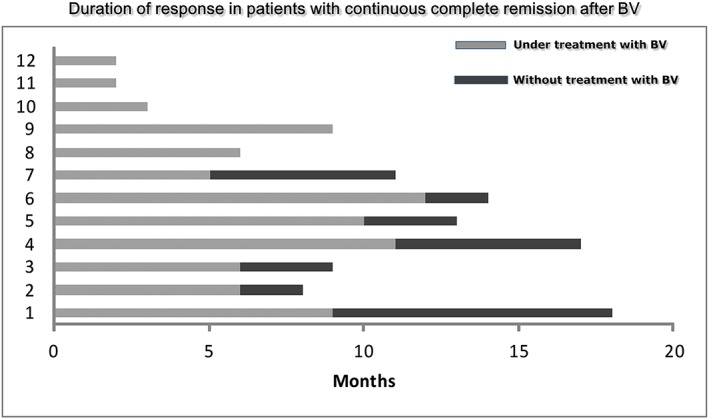
Outcome of complete responders to brentuximab vedotin (BV)

In total, 22 of 95 patients achieved CR after BV. Ten of 22 CRs relapsed at a median time of 9 months (range, 8‐20) after the initiation of BV. The median number of BV cycles administered to this group of patients was 10 (range, 8‐16). Two complete responders received 10 and 16 BV cycles and underwent allo‐SCT thereafter. Five patients completed a median number of 15 BV cycles (range, 9‐16) and remain in CR with no further treatment at a median follow‐up of 13 months (range, 8‐17). Finally, the remaining 5 CRs are still on BV after a median of 3 months (range, 2‐9). Figure 4 depicts the outcome the 12 patients who remain in CR.

## 4 DISCUSSION

This retrospective multicenter Greek study confirms that BV is effective in everyday clinical practice, outside clinical trials.

Our series is the second largest one compared to other national reported experiences.^14^, ^15^, ^16^, ^17^, ^18^ Our patients' characteristics were comparable to the ones among the other series. The Asian series had a higher percentage of patients with advanced clinical stage both at diagnosis and before BV and a higher proportion of patients with B‐symptoms prior to BV initiation.^14^ The Italian and Turkish series reported a higher percentage of patients being refractory to the most recent treatment before BV.^15^, ^16^ Our study included a relatively lower percentage of patients with extranodal involvement at BV initiation.

Our results are in accordance with the other national experience studies with an ORR of 61% including 23% CR.^10^, ^14^, ^15^, ^16^, ^17^, ^18^ In our series, PD was relatively common (27%) in concordance to the Turkish and the French series who also reported a PD rate of 29% and 28%, respectively.^16^, ^18^ Notably, in the pivotal phase II trial and the Asian series, who both reported extremely low rates of PD, response was assessed frequently.^10^, ^14^ Since responses are observed early in the course of treatment, it is likely that frequent response assessments may catch early, short‐living responses, thus minimizing the rate of PD primarily. This is further suggested by the fact that PFS is more or less similar across studies. Our study reflects real life, where response assessments are not prospectively defined and are performed less frequently. Even so, our results are in agreement with the published literature regarding the rapidity of response. We observed best response at a median time of 4 cycles that coincided with the most frequently applied cycle of first disease evaluation. The same observation was made by the French investigators.^18^ In the Italian series, they also observed the best response after the 3rd compared with the 8th cycle.^15^ The retrospective nature of the study and the fact that response assessment was done according to the treating physicians represent a limitation. Thus, response rates should be viewed with caution in this setting, while PFS is a more realistic endpoint.

With the aforementioned limitations, we found that chemosensitivity to the most recent treatment before BV was an independent favorable factor for response achievement. The number of previous regimens and older age (>60 y) were identified as significant factors for response by the Turkish^16^ and French^18^ studies.

Regarding PFS, there is agreement that response to BV is a favorable factor.^10^, ^14^, ^16^, ^17^ In our analysis, there was no significant difference between CR and PR, while PFS was dramatically inferior for nonresponders. This finding is different from the one from the pivotal study,^10^ where CRs had a significantly better outcome compared with PRs. This difference might reflect the poor discrimination between CR and PR outside the clinical trial setting. In addition, the German experience identified primary refractory disease/early relapse and refractoriness to the most recent treatment as additional prognostic factors for PFS.^17^ In our series, refractoriness to the most recent treatment was significant in the univariate analysis, but was obscured in the multivariate setting by response to BV and bulky disease. Our study is the only one denoting the poor prognostic significance of bulky disease at the time of BV initiation for PFS. Bulk proved to be significant for OS as well, along with refractoriness to most recent therapy before BV and the presence of B‐symptoms. This observation is similar to the one of the pivotal trial: The authors found that the sum of the products of the longest perpendicular dimensions of the previously identified dominant lymph node masses was an independent factor for OS, along with age and performance status.^11^

Another issue that has not been clarified yet, is the need for consolidation with allo‐SCT in BV responders. It is clear that patients respond rapidly to BV, crudely after 4 cycles. The French investigators revealed that ORR at the end of BV treatment cycles was dramatically lower than the response after a median of 4 cycles, with PD increasing from 28% to 54%.^18^ These data indicate that the decision to proceed to allo‐SCT should be taken early, since the majority of responders will most likely loose their response to BV during subsequent cycles. The French data are in favor of consolidation of BV responders: Among 145 responders, the 54 patients who received allo‐SCT as consolidation had a significantly longer PFS (median, 18.8 mo) compared with the 91 patients without transplant (median, 8.7 mo).^18^ In our study, the majority of patients who underwent allo‐SCT after BV were alive without progression in contrast to the ones who either received subsequent chemotherapy or those who received no further treatment. Brentuximab vedotin as a bridge to allo‐SCT represents an ideal agent with limited toxicity and most likely improves outcome after allo‐SCT.^19^

The question whether all CRs to BV need consolidation with an allo‐SCT remains unanswered. In the pivotal study, 9 of 34 (26%) complete responders were still in remission after BV without any further therapy at 5 years of follow‐up,^11^, ^20^ indicating that a small proportion of patients failing autograft may achieve long‐lasting remissions with BV. However, this group of patients had inferior PFS compared to the ones who underwent an allo‐SCT.^11^, ^20^ Similarly, in our set of patients, 5 of 22 complete responders (23%) remain in CR after BV with no further treatment. The follow‐up time of the complete responders in the present analysis is short, and no further statements can be made regarding their long‐term outcome. However, it should be noted that in the pivotal study, the vast majority of progressions had occurred by the end of the second year. This observation suggests that maintaining the CR status by our 5 complete responders may not be unlikely.

Another interesting issue is the use of BV as a bridge to ASCT for patients who are in chemorefractory. Published data are promising in this matter.^21^, ^22^, ^23^, ^24^ In our patient population, 20 patients were treated with BV before ASCT due to chemorefractoriness and were intended to proceed to ASCT. Among them, only 5 had an objective response, and finally 10 patients underwent ASCT. The combination of BV with chemotherapy, such as ESHAP (etoposide, methylprednisolone, high‐dose cytarabine, cis‐platinum),^25^ bendamustine,^26^ or gemcitabine/vinorelbine/liposomal doxorubicin^27^ might prove more efficacious as a bridge to ASCT.

## 5 CONCLUSION

Brentuximab vedotin is an effective treatment for R/R HL patients after failure of ASCT, not only within clinical trials but also in everyday clinical practice. The decision for further consolidation with a transplant should be taken early during treatment. Whether BV can cure a fraction of patients remains to be seen.
